# Virological and Immunological Status of the People Living with HIV/AIDS Undergoing ART Treatment in Nepal

**DOI:** 10.1155/2016/6817325

**Published:** 2016-07-28

**Authors:** Chet Raj Ojha, Geeta Shakya, Shyam Prakash Dumre

**Affiliations:** ^1^National Public Health Laboratory, Teku, Kathmandu 44600, Nepal; ^2^Florida International University, College of Medicine, Miami, FL, USA; ^3^World Health Organization, Pulchowk, Lalitpur 44700, Nepal

## Abstract

Antiretroviral therapy (ART) has increased the life span of the people living with HIV (PLHIV), but their virological and immunological outcomes are not well documented in Nepal. The study was conducted at a tertiary care center including 826 HIV-1 seropositive individuals undergoing ART for at least six months. Plasma viral load (HIV-1 RNA) was detected by Real Time PCR and CD4^+^ T-lymphocyte (CD4^+^) counts were estimated by flow cytometry. The mean CD4^+^ count of patients was 501 (95% CI = 325–579) cells/cumm, but about 35% of patients had CD4^+^ T cell counts below 350 cells/cumm. With increasing age, average CD4^+^ count was found to be decreasing (*p* = 0.005). Of the total cases, 82 (9.92%) were found to have virological failure (viral load: >1000 copies/ml). Tenofovir/Lamivudine/Efavirenz (TDF/3TC/EFV), the frequently used ART regimen in Nepal, showed virological failure in 11.34% and immunological failure in 37.17% of patients. Virological failure rate was higher among children < 15 years (14.5%) (*p* = 0.03); however, no association was observed between ART outcomes and gender or route of transmission. The study suggests there are still some chances of virological and immunological failures despite the success of highly active ART (HAART).

## 1. Background

The epidemic of human immunodeficiency virus type 1 (HIV-1) in Nepal is dynamic and concentrated among key populations at higher risk like people who inject drug (PWID), men who have sex with men (MSM) and transgender people, sex workers (SW), and male labor migrants [1]. In 2015, estimated HIV prevalence was 0.2% with estimated cases and reported cases being 39,249 and 26,702, respectively [2]; however, the prevalence data of HIV drug resistance has not yet been available in Nepal. According to recent data, 11089 people living with HIV (PLHIV) were on ART. Of them, 8003 were on regular first-line regimens, 2944 on substituted first-line regimens, and 142 on second-line regimens. Until July of 2015, 2089 cases of AIDS related death have been reported [2].

The primary goal of ART is to suppress HIV-1 RNA lower than the detection level (LDL) of the assay within six months on treatment and restore immunologic function, to reduce morbidity and mortality, to reduce vertical transmission, and to improve overall quality of life [3]. However, there are still unresolved problems including early mortality, incomplete responses, variations in ART outcomes, lack of universal consensus to define treatment failures and time to start ART, drug resistance, and loss to follow-ups [4]. Though HIV-1 RNA testing is the gold standard to monitor patients on ART, due to costs and technical demands needed for it, CD4^+^ T cell measurements are recommended for resource-poor settings [5]. Due to the lack of HIV-1 RNA monitoring in resource-poor settings, patients from these areas are supposed to continue on first-line ART until virological failure progresses to a 50% decrease in CD4^+^ T cell count (immunologic failure) or the recurrence of symptomatic HIV disease (clinical failure). Even then, clinicians may delay switching to second-line therapy, due to the limited availability of second-line medications and the poor specificity of CD4^+^ T cell counts and clinical symptoms for predicting virological failure [6].

Plasma HIV-1 RNA (viral load) testing quantifies the HIV viral burden in the plasma. The viral load is a standard tool used to monitor treatment response in patients taking ART and, in conjunction with the CD4^+^ T cell count, to assess HIV progression. In some situations, viral load may factor into decisions to initiate or change ART [7]. Studies have shown that patients who have high plasma viral loads have an increased risk of progression to symptomatic disease and AIDS compared with patients who have low or undetectable levels [8].

In Nepal, ART service was established in 2004 and viral load facility was started in 2009 through National Public Health Laboratory (NPHL). Phased scale-up has been planned to efficiently and successfully expand viral load testing services, taking into account the targets for enrollment of PLHIV into ART program. Based on the recommendations of Consolidated Treatment Guidelines of World Health Organization (WHO) and National Consolidated guideline on HIV prevention, treatment, and care, Nepal is at the incipient stage of HIV drug resistance and monitoring of early warning indicators [9]. Considering the expanding ART services in the country including the drug resistance surveillance, this study was conducted to establish baseline information on virological and immunological status of the PLHIV receiving ART for more than six months from various regions of Nepal, so that it could facilitate the drug resistance surveillance across the country by providing the insight into the outcome of ART.

## 2. Methodology

### 2.1. Study Design

A descriptive cross-sectional study was conducted at National Public Health Laboratory from November 2013 to June 2015. Patients from four major ART service centers of Nepal, Teku ART Center, Kathmandu; Western Regional Hospital ART Center, Pokhara; Chitwan Hospital ART Center, Chitwan; and Lumbini Zonal Hospital ART Center, Butwal, were enrolled.

### 2.2. Ethical Approval and Patient Recruitment

Ethical approval of the study protocol was obtained from Nepal Health Research Council and 826 patients were recruited after obtaining written informed consent. HIV infected individuals under ART for a minimum of six months (referred to NPHL and other sites for HIV viral load testing) and meeting the study criteria were enrolled.

### 2.3. Patient Data Collection

A standard data collection questionnaire was completed for each patient prior to sample collection. Patient's data including age, sex, ART sites, duration of ART treatment, and ART regimens used were collected at the sample collection site. The data were verified by using the ART center database maintained at ART sites as well as NPHL.

### 2.4. Sample Collection and Preparation

Blood sample (5 mL) was collected by registered laboratory technicians/technologists/nurses using BD K3EDTA Vacutainers and disposable needles. The sample was divided into two separate vials: one for CD4^+^ T cell count and the other for HIV-1 viral load testing. For the latter, the tube with whole blood was centrifuged at 1500 RPM for 10 min, and plasma was separated, aliquoted, and stored at −20°C/−80°C. Plasma samples separated in the peripheral centers were stored at −20°C and were transported to NPHL on the same day of sample collection, where the plasma samples were stored at −80°C until analysis.

### 2.5. Viral RNA Extraction and Plasma Viral Load Quantification

HIV-1 RNA was extracted by using QIAamp Viral RNA Mini Kit (QIAGEN GmbH, Hilden, Germany) following the manufacturer's protocol. The eluted RNA was stored at −80°C until RNA quantification. HIV-1 RNA was quantified by Real Time PCR using artus HI Virus-1 RG RT-PCR Kit (QIAGEN GmbH, Hilden, Germany) and Corbett Rotor-Gene 6000 Real Time PCR system. The PCR conditions were set for denaturation 95°C/30 seconds, annealing 50°C/60 seconds, and elongation 72°C/30 seconds. A range of standards (10, 100, 1,000, and 10,000 IU/*μ*L) provided with the kits were used to develop standard curve for the quantification of the viral load copies per mL. Internal control was used to control the RNA isolation procedure and to check for possible PCR inhibition.

### 2.6. CD4^+^ T-Lymphocyte Estimation by Flow Cytometry

The enumeration of CD4^+^ T lymphocytes was carried out by flow cytometry (FACS Calibur, BD Biosciences, San Jose, CA, USA) using the anticoagulated whole blood following the manufacturer's protocol. Tru-COUNT reagent containing monoclonal antibodies for CD3, CD45, and CD4 labelled with fluorescent dyes (PE, PerCP, and FITC, resp.) was mixed with anticoagulated whole blood and red blood cells were lysed with lysing solution before enumeration. BD MultiSET software (BD Biosciences) was used to determine the absolute CD4^+^ T cell counts.

### 2.7. Statistical Analysis

All the data were recorded in Microsoft Excel 2013. The statistical analysis was carried out by using SPSS 17.0 software and “Openepi” online software. CD4^+^ T cell count < 350/cumm and viral load > 1000 copies/mL after six months of ART treatment were considered as cutoff for immunological and virological failures, respectively. The outcome was analyzed with respect to age, sex, route of transmission of HIV infection, and duration of ART by using Chi-square test and ANOVA.

## 3. Results

**Figure 1:**
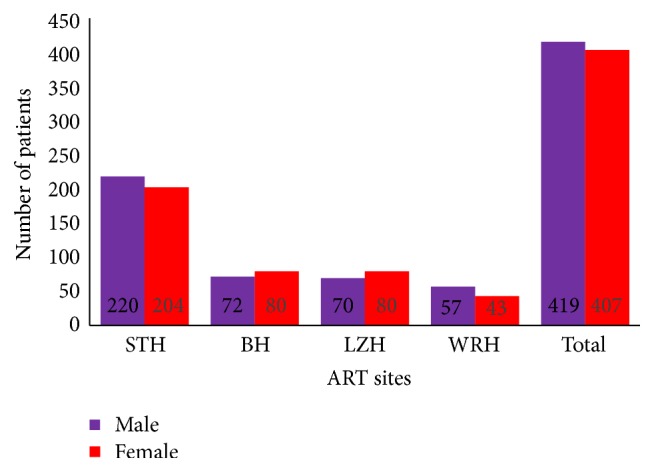
Sample distribution according to sex and referral sites. BH: Bharatpur Hospital, LZH: Lumbini Zonal Hospital, STH: Sukraraj Tropical Hospital, and WRH: Western Regional Hospital. STH is the largest ART service site in Nepal.

The study comprised a total of 826 PLHIV under ART for at least six months from different ART referral sites of the country; out of them 419 were males and 407 were females with male-to-female ratio of 1.03. The mean time duration of ART treatment was 59.7 months. The patients were representative of almost all regions of the country. The number of samples collected from the referral sites is shown in Figure 1.

### 3.1. Immunological Status of the PLHIV in Nepal

**Figure 2:**
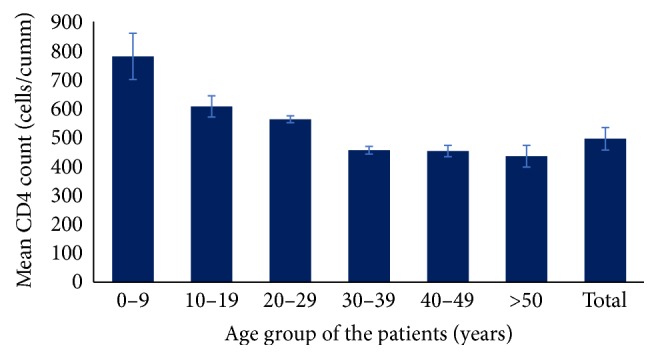
Mean CD4 count of the patients after a duration of six months or more of ART treatment. Error bar shows the standard error of mean (SEM).

Overall immunological status of the patients after taking ART for at least six months was above the threshold line recommended for ART startup, that is, 500 cells/cumm (mean CD4^+^ T cell count = 501 cells/cumm; 95% confidence interval (CI) = 325–579 cells/cumm). With increasing age, the average CD4^+^ T cell count was found to be decreasing (ANOVA, *p* = 0.0005). Average CD4^+^ T cell count was 789 cells/cumm (95% CI; 630–947) in children below 15 years and 440 cells/cumm (95% CI; 365–514) in PLHIV more than 15 years (Figure 2).

### 3.2. Virological Outcomes of the ART in PLHIV in Nepal

**Figure 3:**
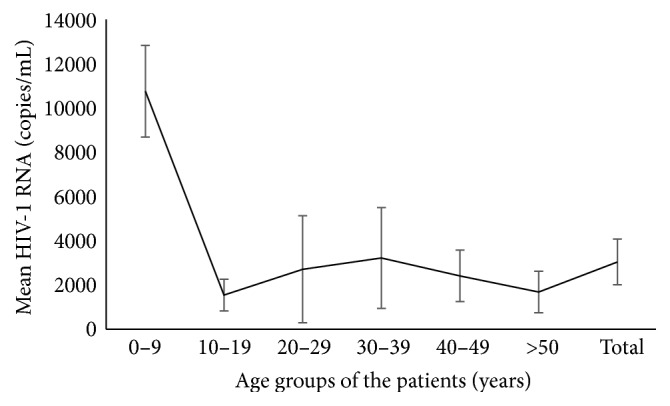
Mean viral load of patients (of different age groups) after six months or more of ART treatment. Error bar shows the standard error of mean (SEM).

Out of total 826 cases, 744 patients were found to have suppressed (<1000 copies/mL) HIV-1 viral RNA level (viral load), whereas 82 (9.92%) patients had shown virological failure (>1000 copies/mL). 129 (15.6% of total) patients had detectable viral load copies which were still below the threshold considered for virological failure. The average HIV-1 viral load was the highest (10,794 copies/mL) in the age group of 0–9 years (ANOVA, *p* = 0.628 by between-group analysis) though statistically insignificant (Figure 3). In the children below 15 years, the frequency of virological failure was slightly higher (14.5%) than the overall rate (Table 1). There was no significant association between virological outcomes in different sex of patients and routes of transmission (Table 1).

### 3.3. Correlation between Virological and Immunological Status of Patients under ART

**Figure 4:**
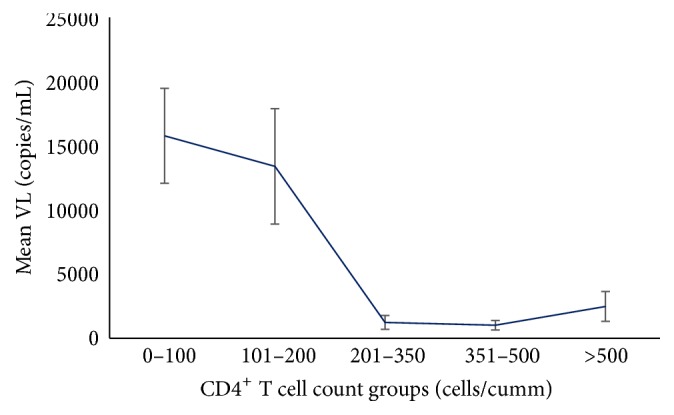
Correlation between CD4^+^ T cell counts and HIV-1 viral load. The mean viral load copies/mL for different CD4 count groups are plotted in the graph. Inverse relationship was observed; with increasing CD4 counts, viral load was found to be decreasing. Error bar showing the SEM.

We found no significant correlation between virological and immunological status of the patients based on absolute means comparison (*p* = 0.72). To address the fluctuating values of viral load, we compared different categories of CD4^+^ T cell levels and viral load levels, where we observed significant association between virological and immunological parameters (*p* = 0.028) with reciprocal relationship between them (Tables 2 and 3; Figure 4).

### 3.4. Response of Commonly Used ART Regimen among PLHIV in Nepal

**Figure 5:**
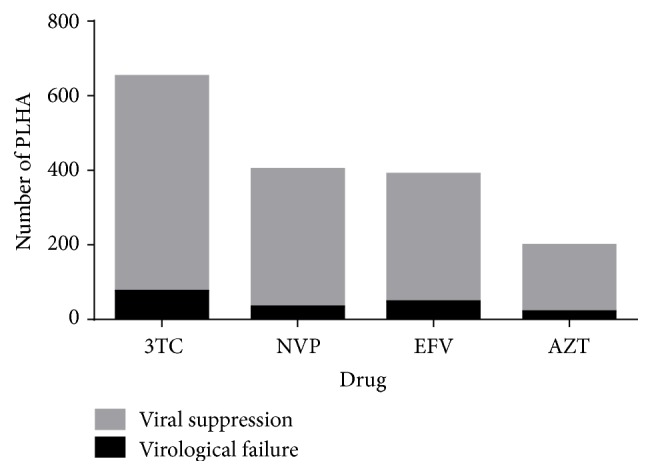
Comparison of selected common drugs used for ART in Nepal. 3TC: Lamivudine; NVP: Nevirapine; EFV: Efavirenz; AZT: Zidovudine. Nevirapine based regimens were found to suppress viral load more effectively in comparison to Efavirenz based regimens (*p* = 0.04).

**Figure 6:**
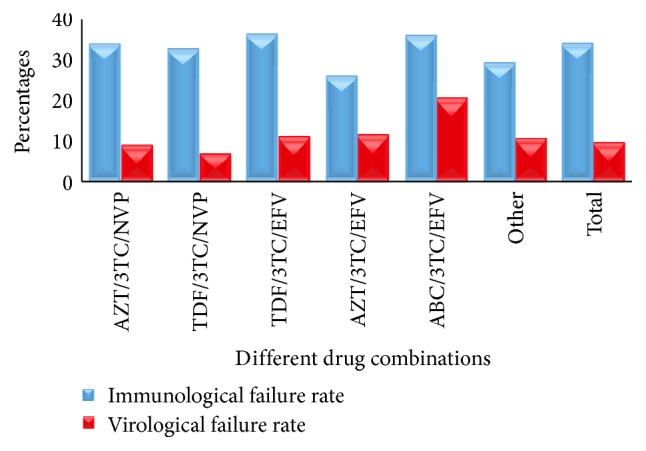
Virological and immunological failure rates with different ART regimes. The blue bar shows the percentage of patients treated with given regimens and having reported to have CD4^+^ T cell counts less than 350 cells/cumm. CD4^+^ T cell count less than 350 cells/cumm was considered borderline to determine eligibility for starting the ART before implementation of the new consolidated guideline for treatment of HIV 2013. Red bar shows the number of patients treated with the given regimen shown to have viral load more than 1000 copies/mL. EFV: Efavirenz, 3TC: Lamivudine, NVP: Nevirapine, AZT: Zidovudine, TDF: Tenofovir, and ABC: Abacavir.

We compared the virological and immunological outcomes of different ART regimens currently being prescribed in Nepal (Tables 4 and 5). Efavirenz based regimens were found less effective than Nevirapine based regimens for virological suppression (Figure 5). Among the common ART combination, Tenofovir/Lamivudine/Nevirapine (TDF/3TC/NVP) was found to be most effective in terms of virological response showing only 7.14% virological failure rate (Table 4). TDF/3TC/EFV, the most commonly used ART regimen in Nepal, had 11.34% virological and 37.02% immunological failure rates (Figure 6). ABC/3TC/EFV was found to be the least effective in terms of both immunological and virological response (Tables 4 and 5; Figure 6).

## 4. Discussion and Conclusion

Since the inception of ART for HIV treatment in 2004, there has been a substantial increment of people on ART and improvement of their health status [10]. With this, the potential for widespread emergence and transmission of HIV drug resistance has been a major concern. HIV drug resistance surveillance program has been integrated into Nepal's national HIV program to emphasize the core elements of Early Warning Indictors (EWI) monitoring and conduct HIV drug resistance survey throughout the country from 2014 to 2020 [11]. The findings of this study give an insight into the situation of people living with HIV (PLHIV) in Nepal in terms of immunological and virological status after ART.

The ART regimens used in Nepal had shown good response in reducing the HIV-1 viral load. The failure rate observed in our study was concordant with the various studies conducted in other countries [12–15]. The overall 9.92% of virological failure may be attributed to various factors like poor adherence to the drug, timely unavailability of drugs, difference in baseline viral load and CD4 counts, HIV transmission route, duration of treatment, nutritional status, and support to the patients [13, 16]. We analyzed and compared the virological and immunological responses in different age, sex, and route of transmission. The data showed that children were more susceptible to the virological failures (*p* = 0.03) compared to adults. However, no significant difference was observed in terms of gender and route of transmission. The immunological recovery was observed in 62.98% to 73.53% of the total study population depending upon the combination of regimens used. Similarly, virological suppression was manifested in 78.95% to 92.86% of the patients. Out of different regimens, TDF/3TC/NVP and AZT/3TC/NVP had better immunological and virological response among Nepalese PLHIV. These results are concordant with Chinese population, where 12.1% failure rate was observed with 3TC combined drugs [13].

Our study showed better outcome of Nevirapine based regimen than Efavirenz based regimen (*p* = 0.04) in line with study results from India [17]. Keeping in view the similar nature of HIV epidemics, social cultural, and geographical similarities between Nepal and India, this comparison seems realistic. So, we can infer from these two studies that Nevirapine based combinations are either better or equivalent to the Efavirenz based regimens (at least in our setting) which are relatively expensive [17]. Although a separate study in Botswana showed better response with Efavirenz based drugs than Nevirapine based drugs [18], difference might be due to discrepancy in prevalence of HIV-1 subtypes in Nepal and Botswana. We did not find significant effect of duration of ART on the virological and immunological outcomes, which was in line with a study conducted in India [19].

There are some limitations in our study; we could not measure the baseline values, the study was conducted in short duration (long term impact needs longer prospective study), drug resistance testing facility has not been yet available in the country, the patient's information was taken from the records retrospectively, and the failure rates were determined based on a single point testing of CD4^+^ T cell and viral load after six months of ART treatment. Despite these limitations, this is the first attempt in Nepal to explore and extract baseline information on the immunological and virological status of Nepalese patients under ART. This information will certainly be useful for the HIV/ART programs in the country. Moreover, we emphasize comprehensive prospective studies including baseline survey, clinical situation of patients, drug resistance testing, and adherence to ART in Nepal.

## Figures and Tables

**Table 1:** Virological outcome of patients under ART based on age, sex, and route of transmission. Compared to adults, more numbers of children below 15 years were found to have virological failure.

Category	Total cases	Virological failure (%)
*Children versus adult *(*p* = 0.03)		
Children < 15 years	96	14 (14.5)
Adults	730	68 (9.3)

*Sex difference *(*p* = 0.18)		
Male	419	46 (10.9)
Female	407	36 (8.8)

*Route of transmission *(*p* = 0.4)		
Intravenous	70	6 (8.5)
Percutaneous	756	76 (10)

**Table 2:** Relationship between virological and immunological status of the patients under ART. The mean CD4^+^ T cell counts for patients with different viral load levels were compared and the difference was found statistically insignificant (Pearson correlation, *p* = 0.172).

Viral load group (copies/mL)	Number of patients (%)	Mean CD4^+^ T cell count (95% CI), cells/cumm
<400	615 (74.46%)	510 (485–536)
400–1000	129 (15.61%)	470 (424–516)
>1000	82 (9.93%)	480 (395–565)

**Table 3:** Virological status in different CD4^+^ T cell count groups. The number and the percentages of the patients out of total patients in the designated CD4^+^ T cell count groups. With increasing CD4^+^ T cell count, the percentage of patients with virological suppression was reported to be increasing (Pearson correlation, *p* = 0.028). VL: viral load.

CD4 count cells/cumm	Number of patients in the group	Number of patients with		
		VL < 400 copies/mL (%)	VL = 400–1000 copies/mL (%)	VL > 1000 copies/mL (%)
0–100	26	14 (53.8%)	5 (19.23%)	7 (26.9%)
101–200	61	38 (62.3%)	13 (21.31%)	10 (16.39%)
201–350	199	150 (75.4%)	32 (16.08%)	17 (8.54%)
351–500	212	162 (76.4%)	28 (13.20%)	22 (10.37%)
>500	328	251 (76.5%)	51 (15.54%)	26 (7.92%)

Total	826	615 (74.46%)	129 (15.61%)	82 (9.93%)

**Table 4:** Virological status of the patients under different ART regimens. The table shows the number of patients treated with different ART regimens and found to have different levels of viral load (detection limit of the assay was 400 copies/mL). VL: viral load. EFV: Efavirenz, 3TC: Lamivudine, NVP: Nevirapine, AZT: Zidovudine, and TDF: Tenofovir.

ART regimens	Number of patients with				VL failure rate (%)
	VL < 400 copies/mL	VL = 400–1000 copies/mL	VL > 1000 copies/mL	Total	
AZT/3TC/NVP	111	37	15	163	9.2
TDF/3TC/NVP	206	15	17	238	7.14
TDF/3TC/EFV	232	65	38	335	11.34
AZT/3TC/EFV	26	4	4	34	11.76
ABC/3TC/EFV	11	4	4	19	21.05
Other	29	4	4	37	10.81

*Total*	*615*	*129*	*82*	*826*	*9.92*

**Table 5:** Immunological status of the patients under different ART regimens in Nepal. The table shows the number of patients treated with different ART regimens and having designated CD4^+^ T cell count groups. In the parentheses are the percentages out of total patients with the particular regimens.

ART regimen	Total cases	Number and percentage of patients with given CD4^+^ T cell count (cells/cumm)					
		<100	100–200	201–350	>350–500	>500	<350
AZT/3TC/NVP	163	4 (2.45%)	11 (6.75%)	41 (25.15%)	35 (21.74%)	72 (44.17%)	56 (34.36%)
TDF/3TC/NVP	238	7 (2.94%)	18 (7.56%)	54 (22.68%)	61 (25.63%)	98 (41.18%)	79 (33.2%)
TDF/3TC/EFV	335	13 (3.88%)	24 (7.16%)	87 (25.97%)	95 (28.36%)	116 (34.63%)	124 (37.02%)
AZT/3TC/EFV	34	0	2 (5.88%)	7 (20.59%)	9 (26.47%)	16 (47.06%)	9 (26.47%)
ABC/3TC/EFV	19	0	3 (15.79%)	4 (21.05%)	3 (15.79%)	9 (47.37%)	7 (36.84%)
Other	37	2 (5.41%)	3 (8.11%)	6 (16.22%)	9 (24.32%)	17 (45.95%)	11 (29.73%)

Total	826	26 (3.14%)	61 (7.38%)	199 (24.09%)	212 (26.67%)	256 (30.99%)	286 (34.62%)

## Abbreviations

3TC: Lamivudine
ABC: Abacavir
AIDS: Acquired immune deficiency syndrome
ART: Antiretroviral therapy
AZT: Zidovudine
CD: Cluster of differentiation
D4T: Stavudine
EFV: Efavirenz
MSM: Men who have sex with men
NCASC: National Center for AIDS and STD Control
NPHL: National Public Health Laboratory
NVP: Nevirapine
PWID: People who inject drug
RT-PCR: Real time polymerase chain reaction
TDF: Tenofovir
PLHIV: People living with HIV.
